# The network configuration in Parkinsonian state compensates network activity change caused by loss of dopamine

**DOI:** 10.14814/phy2.15612

**Published:** 2023-02-17

**Authors:** Katsunori Kitano

**Affiliations:** ^1^ Department of Information Science and Engineering Ritsumeikan University Kusatsu Japan

**Keywords:** dopamine, globus pallidus, Parkinson diseases, subthalamic nucleus

## Abstract

Parkinson's disease is a movement disorder caused by dopamine depletion in the basal ganglia. Neural activity of the subthalamic nucleus (STN) and globus pallidus externus (GPe) in the basal ganglia are closely related to motor symptoms of Parkinson's disease. However, the pathogenesis of the disease and the transition from the normal state to the pathological state have yet to be elucidated. The functional organization of the GPe is gaining attention due to the recent finding that it consists of two distinct cell populations, namely prototypic GPe neurons and arkypallidal neurons. Identifying connectivity structures between these cell populations, as well as STN neurons, in relation to the dependence of the network activity on the dopaminergic effects is vital. In the present study, using a computational model of the STN‐GPe network, we explored biologically plausible connectivity structures between these cell populations. We evaluated the experimentally reported neural activities of these cell types to elucidate the effects of dopaminergic modulation and changes caused by chronic dopamine depletion, such as strengthened connections in the neural activity of the STN‐GPe network. Our results indicate that the arkypallidal neurons receive cortical inputs separately from the source for prototypic and STN neurons, suggesting that arkypallidal neurons might be responsible for an additional pathway with the cortex. Furthermore, changes caused by chronic dopamine depletion compensate for the loss of dopaminergic modulation. Changes caused by dopamine depletion itself likely induce the pathological activity observed in patients with Parkinson's disease. However, such changes counteract those of firing rates caused by loss of dopaminergic modulation. In addition, we observed that the STN‐GPe tends to exhibit activity with pathological characteristics as side effects.

## Graphical abstract

Using a STN‐GPe network model that incorporates physiological properties of cells and synapses in the network, we searched for connectivity between cell populations that would satisfy the firing characteristics observed experimentally in normal and pathological conditions. Numerical simulations using the resulting model showed that changes due to chronic dopamine depletion compensate for the loss of dopamine regulation.

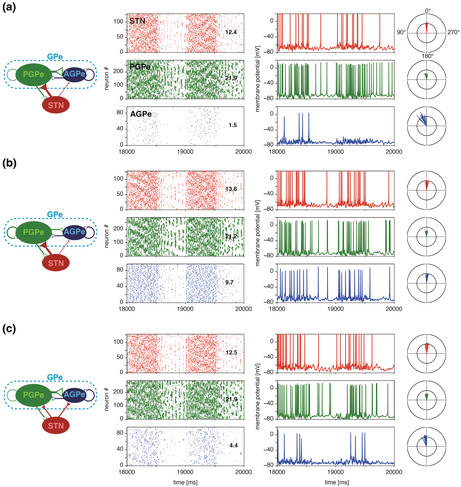

## 1 INTRODUCTION

The glutamatergic subthalamic nucleus (STN) and the γ‐aminobutyric acid (GABA)ergic external segment of the globus pallidus (GPe) form a reciprocally connected network that regulates information flow in the corticobasal gangliothalamic circuit. The STN‐GPe network exhibits asynchronous, tonic activity under normal conditions and relatively synchronous, phasic (often rhythmic) activity in Parkinson's disease and its experimental models (Mallet et al., 2008; Raz et al., 2000; Urbain et al., 2000). Thus, the activity pattern of the STN‐GPe network is closely correlated with motor function. Indeed, modification of abnormal STN‐GPe activity by high‐frequency electrical stimulation, optogenetic manipulation, or chemogenetic manipulation of the STN or GPe ameliorates motor dysfunction in Parkinsonism.

The intrinsic properties of the STN‐GPe network have been investigated in organotypic culture (Plenz & Kitai, 1999), in ex vivo (Baufreton et al., 2005; Bevan et al., 2002) and in vivo (Nambu et al., 2000; Tachibana et al., 2011) preparations, and computational models (Koelman & Lowery, 2019; Lindahl & Hellgren Kotaleski, 2016; Shouno et al., 2017; Terman et al., 2002). These studies have revealed that reciprocal connections between the STN and GPe are key to the generation of pathological rhythmic network activity. Additionally, recent studies have revealed that the GPe consists of two distinct neuron types: prototypic neurons that project to the downstream basal ganglia, and arkypallidal neurons that only project to the striatum (Abdi et al., 2015; Mallet et al., 2012, 2019). Although previous studies have incorporated these neuron types into computational models (Nevado‐Holgado et al., 2014), the detailed structures of the STN‐GPe network that support normal and pathological activity patterns have not been fully explored.

Given that the loss of dopamine triggers the transition of the STN‐GPe network from normal to pathological activity (Bevan et al., 2002; Nambu et al., 2000; Sharott et al., 2005; Tachibana et al., 2011), it is critical to identify the changes in the network properties that contribute to this transition. STN‐GPe network activity is determined by the dynamic properties of its constituent neurons and synapses and afferent inputs. Dopamine depletion has been suggested to trigger changes in all factors such as cellular properties and synaptic transmission (Baufreton & Bevan, 2008; Cooper & Stanford, 2001; Hernández et al., 2006; Miguelez et al., 2012). Nevertheless, how changes in these factors cause the transition to pathological activity remains unclear.

To address these issues, we constructed a computational model of the STN‐GPe network. We first constructed single neuron models of STN and GPe neurons based on the membrane properties previously reported by electrophysiological studies. Using these model neurons, we explored network architectures that could reproduce the network activity observed during cortical slow wave activity (SWA) and cortical activation (ACT). We then introduced parameter changes caused by dopamine depletion to determine how they contribute to pathological network activity.

## 2 MATERIALS AND METHODS

### 2.1 Neuron and synapse modeling

#### 2.1.1 Neuron models and parameter exploration

The models of the STN, prototypic external globus pallidus (PGPe), and arkypallidal (AGPe) neurons were derived from modifications of the model by Fujita et al. (2012), which is a conductance‐based, single‐compartment model. The membrane potential of the STN neuron model obeyed the following equation:

$$\begin{array}{c} c_{m}dV/dt=–I_{\mathrm{NaF}}–I_{\mathrm{NaP}}–I_{\text{Kv2}}–I_{\text{Kv3}}–I_{\mathrm{CaH}}–I_{\mathrm{CaT}}\\ \;–I_{\mathrm{HCN}}–I_{\mathrm{SK}}–I_{\text{leak}}+I_{\mathrm{app}}/A_{\mathrm{eff}}, \end{array}$$

where *I*_Xs_ and *I*_app_ represent the ionic and injected currents, respectively; *A*_eff_ the effective membrane area for a single‐compartment model. The kinetics of the T‐type calcium current, *I*_CaT_, were obtained from the model by Atherton et al. (2010). Similarly, the equation for the GP neurons was as follows:

$$\begin{array}{c} c_{m}\;dV/dt=–I_{\mathrm{NaF}}–I_{\mathrm{NaP}}–I_{\text{Kv2}}–I_{\text{Kv3}}–I_{\mathrm{Kv}4f}–I_{\mathrm{Kv}4s}\\ \;–I_{\text{KCNQ}}–I_{\mathrm{CaH}}–I_{\mathrm{HCN}}–I_{\mathrm{SK}}–I_{\text{leak}}+I_{\mathrm{app}}/A_{\mathrm{eff}}. \end{array}$$

The source codes of the proposed models were uploaded to ModelDB (https://senselab.med.yale.edu/modeldb/).

The kinetics of the gate variables of the ion channels were not modified from the previous model by Fujita et al. (2012). The model parameters, maximal conductances of the ionic channels, and effective membrane areas were explored to reproduce multiple firing features. Using electrophysiological data as a guide, each feature was quantitatively characterized as the mean and standard deviation (*μ* ± *σ*) (Table 1). When the target value of the *i*th feature was defined as *μ*_*i*_ ± *σ*_*i*_, the feature of the model neuron was evaluated using the score *z*_*i*_ = (*v*_*i*_–*μ*_*i*_)/*σ*_*i*_, where *v*_*i*_ is the value of the *i*th feature of the model neuron. Thus, the cost function to be minimized was found as the sum of the squared scores:

$$\sum_{i=1}^{N}z_{i}^{2}.$$

To optimize model parameters, the differential evolution algorithm was applied (Storn & Price, 1997).

**TABLE 1:** Properties and parameters of model neurons.

Injected current	Features	Target^a^	Obtained	Score
STN neuron				
0.0 nA	Firing rate (spikes/s)	7.8 ± 2.1	11.8	1.91
	AP threshold (mV)	−54.0 ± 1.4	−47.5	4.66
0.04 nA	Firing rate (spikes/s)	22.5 ± 6.0	34.8	2.04
0.2 nA	Firing rate (spikes/s)	136.4 ± 19.6	88.3	−2.46
−0.05 nA	# of rebound spikes	4.0 ± 1.0	4	0.00
	Minimum V (mV)	−85.0 ± 3.0	−82.7	0.77
	Depolarization (mV)	5.0 ± 0.1	5.0	−0.40
1.0 nA + CaH block	Ratio of firing rate (*f*_w DA_/*f*_w/o DA_)	2.1 ± 0.2	2.24	0.70

*g*_NaF_	*g*_NaP_	*g*_Kv2_	*g*_Kv3_	*g*_CaH_	*g*_CaT_	*g*_HCN_	*g*_SK_	*g*_leak_	*A*_eff_
Obtained parameters									
10.0	0.07	0.0	18.3	0.45	0.19	0.028	0.27	0.048	5477

Injected current	Features	Target^b^	Obtained	Score
GPe prototypic neuron				
0.0 nA	Firing rate (spikes/s)	20.0 ± 2.0	20.3	0.15
	Adaptation	1.0 ± 0.05	1.00	−0.08
	AP threshold (APth) (mV)	−56.6 ± 1.8	−58.7	−1.15
	AHP (mV)	−67.3 ± 2.2	−69.7	−1.11
	APth–AHP (mV)	10.6 ± 1.2	11.1	0.38
0.1 nA	Firing rate (spikes/s)	60.7 ± 7.6	77.2	2.18
	Adaptation	0.64 ± 0.03	0.62	−0.72
	APth–AHP (mV)	9.5 ± 1.1	8.51	−0.90
−0.1 nA	Firing rate after hyperpolarization (spikes/s)	46.6 ± 13.5	23.8	−1.69
	Minimum V (mV)	−100 ± 1.0	−100.0	0.00
	Depolarization (mV)	5.0 ± 0.05	5.0	−0.02

*g*_NaF_	*g*_NaP_	*g*_Kv2_	*g*_Kv3_	*g*_Kv4f_	*g*_Kv4s_	*g*_KCNQ_	*g*_CaH_	*g*_HCN_	*g*_SK_	*g*_leak_	*A*_eff_
Obtained parameters											
1.4	0.74	0.0	50.0	10.0	0.0	0.37	0.53	0.006	0.095	0.037	6000

Injected current	Features	Target^b^	Obtained	Score
GPe arkypallidal neuron				
0.0 nA	Firing rate (spikes/s)	5.0 ± 1.0	6.0	1.00
	Adaptation	1.0 ± 0.05	1.00	−0.08
	AP threshold (APth) (mV)	−55.0 ± 1.8	−55.1	−0.06
	AHP (mV)	−73.3 ± 1.8	−74.6	−0.71
	APth–AHP (mV)	18.2 ± 1.2	19.5	1.06
0.1 nA	Firing rate (spikes/s)	31.5 ± 2.9	31.0	−0.16
	Adaptation	0.78 ± 0.04	0.73	−1.06
	APth–AHP (mV)	10.9 ± 1.0	13.1	2.17
−0.1 nA	Firing rate after hyperpolarization (spikes/s)	9.9 ± 3.2	7.0	−0.91
	Minimum V (mV)	−103 ± 1.0	−98.9	4.06
	Depolarization (mV)	8.0 ± 0.05	7.9	−1.19

*g*_NaF_	*g*_NaP_	*g*_Kv2_	*g*_Kv3_	*g*_Kv4f_	*g*_Kv4s_	*g*_KCNQ_	*g*_CaH_	*g*_HCN_	*g*_SK_	*g*_leak_	*A*_eff_
Obtained parameters											
3.05	0.39	0.38	1.00	10.0	2.66	0.76	0.42	0.02	0.675	0.090	2087

*g*_X_ in mS/cm^2^ and *A*_eff_ in μm^2^.

^a^ Ramanathan et al. (2008).

^b^ Abdi et al. (2015).

#### 2.1.2 Synapse models

AMPA‐mediated synaptic currents from STN to GPe and GABAergic currents from GPe to STN and GPe to GPe have been described with an extended version of the depressing synapse model (Tsodyks & Markram, 1997) because synaptic currents, particularly from GPe to STN, exhibit a highly stochastic nature of neurotransmitter release (Atherton et al., 2013). The synaptic current arising from a synapse was represented by the following equation:

$$I_{\mathrm{syn}}=g_{\mathrm{syn}}e\left(V-E_{\mathrm{syn}}\right),$$

where *e* is the effective ratio of neurotransmission, and *g*_syn_ and *E*_syn_ (syn = AMPA or GABA‐A) the maximal conductance and reversal potentials, respectively. The effective ratio *e* was determined using the following dynamics:

$$\begin{array}{ccc} \frac{du}{dt} & = & -\frac{u}{\tau_{u}}+U\left(1-u\right)\delta \left(t-t_{\mathrm{AP}}\right)\\ \frac{de}{dt} & = & -\frac{e}{\tau_{e}}+r_{\mathrm{rel}}\delta \left(t-t_{\mathrm{AP}}\right)\\ \frac{dr}{dt} & = & \frac{1-r-e}{\tau_{r}}-r_{\mathrm{rel}}\delta \left(t-t_{\mathrm{AP}}\right), \end{array}$$

where *r* and *u* represent the recovered ratio and release probability, respectively. Additionally, *τ*_*e*_, *τ*_*u*_, *τ*_*r*_, and *t*_AP_ are the time constants of inactivation, facilitation, recovery, and timing of action potential, respectively. The kinetic parameters are listed in Table 2. The release ratio *r*_rel_ was a stochastic variable. We defined *r*_v_ as the ratio per vesicle, which indicated the ratio occupied by the number of neurotransmitters packed within a vesicle. Accordingly, we defined the number of vesicles, *n*_v_ as *n*_v_ = [*r*/*r*_v_], where the bracket [*x*] is the maximal integer that is less than *x*. Moreover, *r*_rel_ was represented by stochastic variables, *v*_*i*_:

$$r_{\mathrm{rel}}=\sum_{i=1}^{n_{v}}v_{i},$$

where *v*_*i*_ takes *r*_v_ with the release probability, *u* or 0 with 1–*u*. The ratio, *r*_rel_/*r*_v_ obeys the binominal distribution with the mean *n*_v_ *u*. If *r*_rel_ is replaced by the mean *r*_v_ *n*_v_ *u* ~ r *u*, the model coincides with the original depressing synapse model with deterministic kinetics (Tsodyks & Markram, 1997). This implementation was similar to the model proposed by Loebel et al. (2009). The values of the parameters *r*_v_ and *u* were obtained by minimizing the sum of the squared errors between the experimental and simulated data (reliability and normalized conductance) for all input frequencies (Table 3). The obtained values were *r*_v_ = 0.008 and *u* = 0.1 (Table 3). GPe‐STN synapses exhibit short‐term depression, whereas the dynamics of STN‐GPe synapses vary from depression to facilitation (Hanson & Jaeger, 2002). In our simulation, the STN‐GPe synapses were modeled as a facilitating type.

**TABLE 2:** Parameters of synapse models.

	GPe‐STN	STN‐GPe	Ctx‐STN	Str‐GPe
*τ*_*e*_ (ms)	7.7	3.0	3.0	8.0
*τ*_*r*_ (ms)	17,300	200	800	100
*τ*_*u*_ (ms)	—	100	—	700
*U*	0.1	0.1	0.1	0.14

Abbreviations: Ctx, cortex; GPe, external globus pallidus; STN, subthalamic nucleus; Str, striatum.

**TABLE 3:** Properties of GPe‐STN synapse model.

Frequency (Hz)	Reliability (%)	Normalized conductance (%)
1	100 (91.67 ± 20.41)	67 (66.09 ± 39.36)
10	46 (40.83 ± 32.93)	14 (29.39 ± 29.50)
20	28 (22.08 ± 19.52)	12 (12.71 ± 11.93)
33	18 (16.64 ± 7.89)	11 (9.22 ± 6.74)
100	7.5 (6.4 ± 4.89)	11 (3.44 ± 2.74)

Abbreviations: Mean ± SD in the brackets from Atherton et al. (2013).

In addition to the AMPA‐mediated synaptic currents, NMDA‐mediated currents were implemented in synaptic currents from the STN to the GPe. This type of current was modeled using a first‐order kinetic model (Destexhe et al., 1998).

In the present study, synaptic inputs from the cortex to the STN and GPe, as well as from the striatum to the GPe were implemented as background synaptic inputs, which were modeled as a point‐conductance model (Destexhe et al., 2001). The background synaptic current and conductance dynamics were determined by the following equations:

$$\begin{array}{ccc} I_{\mathrm{syn}}^{X} & = & g_{X}\left(V-E_{X}\right),\\ \frac{dg_{X}}{dt} & = & -\frac{g_{X}-g_{X}^{0}}{\tau_{e}^{X}}+\eta_{X}\left(t\right), \end{array}$$

where *g*_X_^0^, and *η*_X_ (x = Cortex [Ctx] or Striatum [Str]) represent the average conductance, and Gaussian white noise, respectively. When *D*_X_ is the diffusion coefficient, *η*_X_(*t*) satisfied <*η*_X_(t) ≥ 0 and <*η*_X_(t)*η*_X_(s)≥ *D*_X_δ(*t*–*s*). The values of *τ*_e_^X^ represent the decay time constants of the corresponding synapse types. The background synaptic inputs consist of *N*_X_ synapses, each of which was described by the depressing synapse model (Tsodyks & Markram, 1997);

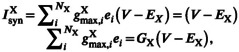

where *G*_X_ represents the total conductance of *N*_X_ synapses. If presynaptic neurons generate Poisson spike trains, then *G*_X_ can be approximated as the point conductance model obeying the following equations:

$$\begin{array}{ccc} G_{X}^{0} & = & g_{\max,X}N_{X}R_{X}\tau_{e}^{X}S_{X},\\ D_{X} & = & g_{\max,X}^{2}N_{X}R_{X}{\left(S_{X}\right)}^{2}, \end{array}$$

$$S_{X}=\frac{\left(1-\exp\left[-1/R_{X}\tau_{r}^{X}\right]\right)}{U_{X}+\left(1-U_{X}\right)\left(1-\exp\left[-1/R_{X}\tau_{r}^{X}\right]\right)\left(1-\exp\left[-1/R_{X}\tau_{u}^{X}\right]\right)},$$

where *g*_max,X_, *N*_X_, and *R*_X_ are the maximal conductance, number of connections to a postsynaptic neuron, and firing rates of presynaptic neurons, respectively. The other parameters were from the model parameters of the synapse type X. If the firing rate is maintained at a certain level, *G*_X_ reaches the steady state of $G_{X}^{0}$ ^0^. The maximal conductance of Ctx‐STN synapses was set to 3.75 nS because the synaptic current evoked by optogenetic stimulation is 150 pA at a holding potential of −40 mV (Chu et al., 2017). Recent studies have reported that cortical neurons send their axons to the GPe (Abecassis et al., 2020; Karube et al., 2019). The synaptic strength from the Ctx to the PGPe was similar to that to the STN, whereas that to the AGPe was 50% larger (Karube et al., 2019). Therefore, we set the maximal conductance for a Ctx‐PGPe synapse and a Ctx‐APGe synapse to 3.75 and 5.63 nS, respectively. The ratios of effective synaptic connections to the PGPe and the AGPe were estimated to make up approximately 40% and 50% of Ctx‐STN connections, respectively (Karube et al., 2019). Thus, the Ctx‐PGPe and Ctx‐AGPe connections were set to 0.4*N*_ctx‐STN_ and 0.5*N*_ctx‐STN_, respectively (*N*_ctx‐STN_ is the number of connections from the cortex to STN). An optogenetic stimulation study showed that PGPe and AGPe neurons receive GABAergic synaptic inputs from indirect‐ and direct‐pathway striatal projection neurons (iSPN and dSPN), respectively (Cui, Du, et al., 2021). Depending on the stimulus intensity, the ratio of inhibitory postsynaptic current (IPSC) amplitudes in the PGPe to those in the AGPe ranged from 8 to 12. Moreover, the strength of the iSPN‐PGPe connection was assumed to be 10 times greater than that of the dSPN‐AGPe connection. The optogenetically evoked current amounts to approximately 200 pA for a voltage difference of 60 mV, yielding a maximal conductance at putative iSPN‐PGPe synapses of 3.33 nS (Miguelez et al., 2012). Therefore, the maximum conductance of the dSPN‐AGPe connection was set to 0.33 nS. The firing rate (*R*_X_) was based on experimental evidence, but the value of the Parkinsonian state was explored as necessary.

We considered rhythmic inputs from the cortex and striatum. In the case of SWA, the time course of the firing rate, *R*_X_, was defined by the following equation:

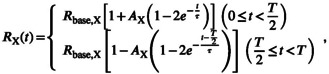

where *R*_base,X_, *A*_X_, *τ*, and *T* are the mean firing rate, amplitude, time constant, and period, respectively. We set *τ* and *T* to 50 ms and 1 s, respectively. *A*_X_ is the parameter to be explored. The firing rate of spontaneous activity of corticostriatal neurons under normal conditions was 1.5 spikes/s (Mallet et al., 2006). Moreover, the firing rates of iSPNs and dSPNs in control rats were 0.34 and 0.31 spikes/s, respectively (Sharott et al., 2017).

For cortical activation (ACT), the firing rate was oscillated with frequency, *f*:

$$R_{X}\left(t\right)=R_{\text{base},X}\left[1+A_{X}\sin\left(2\mathrm{\pi ft}-\phi_{X}\right)\right],$$

where ϕ_X_ represents a phase.

The firing rates of iSPN and dSPN were 0.49 and 1.05 spikes/s, respectively (Sharott et al., 2017). Because no distinct rhythmic component of cortical and striatal activity during ACT was observed in the normal state (Sharott et al., 2017), *A*_Ctx_ and *A*_Str_ were set to 0.

#### 2.1.3 Network modeling

##### Synaptic connections

**FIGURE 1:**
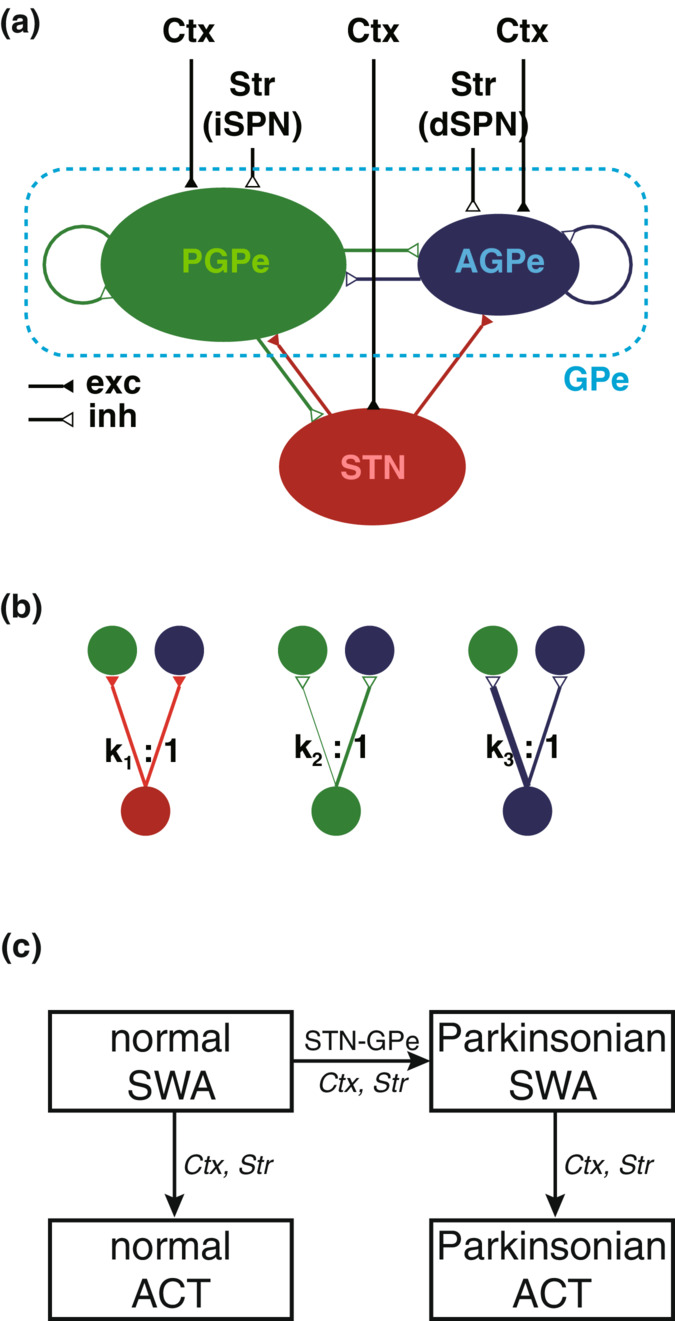
Structure of STN‐GPe network model. (A) STN‐GPe network model. The network consists of STN, PGPe, and AGPe neurons. The inputs from the cortex and striatum are modeled as afferent inputs to the network. (B) Biases of connections to PGPe versus AGPe neurons. The bias parameters *k*_1_, *k*_2_, and *k*_3_ represent the biases of STN‐GPe, PGPe, and AGPe neurons, respectively. The thickness of the arrows indicates the degree of the biases but not the strengths of connections (i.e., synaptic conductance). In the example on STN to GPe connections (left), the arrows of STN‐PGPe and STN‐AGPe connections are the same, suggesting no bias (*k*_1_ = 1). In the example of PGPe‐GPe connections (middle), the arrow of PGPe–PGPe is thin, indicating that the bias is small (*k*_2_ < 1). In contrast, in the example of AGPe–GPe connections, AGPe neurons connect to more AGPe neurons than PGPe ones (*k*_3_ > 1). (C) The procedure of modeling various conditions. At first, the parameters on afferent inputs and connectivity biases were explored so that each cell population satisfies each firing characteristic during normal SWA. The state of the Parkinsonian SWA is obtained by applying known parameter changes, such as strengthened or weakened synaptic connections, and adjusting the parameters concerning the afferent inputs. Normal legends along the arrow indicate that known parameter changes are to be applied, whereas italic legends indicate that the parameters must be adjusted.

The number of neurons in the network model was based on the experimentally estimated number of STN and GPe neurons (in rats). The number of STN neurons was estimated to be 13,600, and that of GPe neurons was 43,700 (Oorschot, 1996). Thus, the number of GPe neurons was approximately three times larger than that of STN neurons. The present model of the STN‐GPe network consisted of 1024 STN neurons (*N*_STN_ = 1024) and 3072 GPe neurons (*N*_GPe_ = 3072). The GPe has been found to consist of two distinct cell populations: PGPe and AGPe neurons (Mallet et al., 2012). Because the number of AGPe neurons was estimated to make up 20%–25% of the GPe, 25% of GPe neurons were defined as AGPe neurons (*N*_AGPe_ = 768), the remainder were defined as PGPe neurons (*N*_PGPe_ = 2304) in the present model (Figure 1A). We then estimated the strength of inter‐ and intra‐nuclei connections based on several experimental studies.

##### GPe‐STN connections

The number of GPe‐STN synapses has been estimated to be 12,000,000 (Baufreton et al., 2009). Thus, the average number of GPe‐STN synapses on a single STN neuron is 12,000,000/13,600 = 882. Additionally, the mean conductance of miniature IPSCs was found to be 0.73 nS, whereas the peak conductance of evoked IPSCs was 6.82 nS (Baufreton et al., 2009). Thus, if 6.82/0.73 = 9.3 synapses from a GPe neuron connect to a single STN neuron, the average number of presynaptic GPe neurons to an STN neuron is 94.8. Here, we set the number of GPe‐STN connections (the number of presynaptic neurons from which a postsynaptic neuron received synaptic inputs) to *N*_GPe‐STN_ = 100. The *g*_GPe‐STN_ was set to 6.82 nS.

##### GPe–GPe connections

The average number of boutons on an axon from a GPe neuron has been estimated to be 581 (Sadek et al., 2007). Moreover, the mean conductance of miniature IPSCs was 0.73 nS, and the peak conductance of evoked IPSCs was 6.67 nS (Miguelez et al., 2012). A recent study showed that the synaptic strength from a PGPe neuron to a PGPe neuron was estimated to be 4.38 nS, whereas that from a PGPe neuron to an AGPe neuron was 6.17 nS (Aristieta et al., 2021). As a result, a single PGPe neuron makes six synapses with a PGPe neuron and eight with an AGPe neuron. On the other hand, the optogenetic stimulation of AGPe neurons showed that the amplitude of the synaptic current by an AGPe–PGPe connection is 20% of that by a PGPe–AGPe connection (Cui, Pamukcu, et al., 2021). However, no data are available regarding AGPe–AGPe connections. Therefore, we assumed that the strength of AGPe–AGPe connections is the same as that of AGPe–PGPe connections. Because optogenetic stimulation of AGPe has no impact on PGPe or AGPe neurons (Aristieta et al., 2021) and the main targets of AGPe innervation are outside the GPe, such as the striatum (Abecassis et al., 2020), only approximately 20% of synapses on the axons of AGPe neurons are assumed to make contact with PGPe or AGPe neurons. When we denote the number of connections received by a PGPe and an AGPe neuron as *n*_PGPe_ and *n*_AGPe_, respectively, these two parameters should satisfy the following relationship: 581 × *N*_PGPe_ = 6 × *n*_PGPe_ × *N*_PGPe_ + 8 × *n*_AGPe_ × *N*_AGPe_ for synapses from PGPe neurons. If we introduce the connection bias parameter *k* that is defined as *n*_PGPe_ = *k n*_AGPe_, then *n*_PGPe_ and *n*_AGPe_ can be expressed in terms of *k*. Additionally, *g*_PGPe‐PGPe_ and *g*_PGPe‐AGPe_ were set to 4.38 and 6.67 nS, respectively, and the *g*_AGPe‐PGPe_ and *g*_AGPe‐AGPe_ were both set to 1.33 nS.

##### STN‐GPe connections

STN neurons that send their axons to the GPe produce 457 (±425) boutons on average (Koshimizu et al., 2013). Thus, the GPe receives approximately 6 million STN‐GPe synapses, and consequently, a single GPe neuron has 142 synapses. Because the conductance of miniature EPSCs has been found to be 0.14–0.18 nS (Hernández et al., 2006) and 0.25–0.30 nS (Chen et al., 2002), we assumed the value to be 0.2 nS. A recent study showed that the synaptic strength from the STN to the PGPe (5.429 nS) is approximately four times stronger than that to the AGPe (1.423 nS) (Aristieta et al., 2021). As a result, 5.429/0.2 ~ 27 synapses from an STN neuron make contact with a single PGPe neuron, whereas an AGPe neuron receives 1.423/0.2 ~ 7 synapses from an STN neuron; accordingly, 457 × *N*_STN_ = 27 × *n*_PGPe_ × *N*_PGPe_ + 7 × *n*_AGPe_ × *N*_AGPe_. As with the GPe–GPe connections, we introduced the connection bias *k* to express the number of connections in terms of *k*. The *g*_STN‐PGPe_ and *g*_STN‐AGPe_ were set to 5.4 and 1.4 nS, respectively.

#### 2.1.4 Network structure

##### Connectivity bias

While the number of connections for a neuron was estimated above, it was based on the results of studies where the two types of globus pallidus neurons were not differentiated. Therefore, how the two types of neurons share intra‐ and inter‐nuclei connections is unknown. To clarify this, we introduced connectivity bias parameters (Figure 1B). For each type of connection, the parameter represents the ratio of afferent connections of a prototypic neuron to those of an arkypallidal neuron. These parameters (*k*_1_, *k*_2_, and *k*_3_) require further exploration.

##### Topographic/non‐topographic connectivity

The primate basal ganglia have been reported to exhibit somatotopic organization (Nambu, 2011; Rodriguez‐Oroz et al., 2001). If the rodent basal ganglia is also characterized by such organization, spatial patterns of inter‐nuclei connections may have functional significance. To introduce this characteristic, we arranged each type of neuron into a one‐dimensional array (Figure 1C). The relative location in the one‐dimensional array was assumed to correspond to specific body parts in somatotopic organization. Here, we define topographic connectivity as a connection pattern in which a neuron in a nucleus makes synaptic contacts selectively with neurons of the other nucleus in the vicinity of the corresponding location (the same relative location in the one‐dimensional array). In the case of non‐topographic connectivity, postsynaptic neurons were randomly selected from all neurons of the other nucleus. In the present study, It is assumed that STN‐PGPe/AGPe and PGPe‐STN connections are topographic, whereas the other connections are random.

#### 2.1.5 Effects of dopamine

##### Dopaminergic modulation under normal conditions

The activation of D2‐like dopamine receptors, presumably on presynaptic terminals, decreases the probability of the release of neurotransmitters in inhibitory GPe‐STN synapses (Baufreton & Bevan, 2008) and excitatory STN‐GPe synapses (Hernández et al., 2006). However, no significant change was observed in GPe–GPe synapses (Miguelez et al., 2012). Additionally, activation of D1‐like dopamine receptors has been found to increase neurotransmitter release (Tecuapetla et al., 2007). Therefore, under normal conditions, we decreased the release probability (*U*) of GPe‐STN, STN‐GPe, and Str(iSPN)‐PGPe synapses by 50% and increased that of Str(dSPN)‐AGPe by 50%.

##### Dopamine‐depleted condition

###### Changes in connection strengths

A significant change found in dopamine‐depleted STN‐GPe circuits was substantial strengthening of the GPe‐STN connections caused by an increase in the number of synapses by 50%–100% (Fan et al., 2012). GPe–GPe connections have also been found to be potentiated by 40% after chronic dopamine depletion (Miguelez et al., 2012). To incorporate these effects into our model, we increased the GABAergic conductance of GPe‐STN and GPe–GPe connections by 50% and 40%, respectively. Regarding the STN‐GPe connections, STN‐PGPe connections are weakened by 30% after dopamine depletion, whereas STN‐AGPe connections are not affected (Pamukcu et al., 2020). Accordingly, we decreased the conductance of STN‐PGPe connections by 30%. Furthermore, because the number of Ctx‐STN connections is reduced by 50%–75% after dopamine depletion (Chu et al., 2017), we decreased the conductance of Ctx‐STN connections by 50%. Currently, no evidence of changes in the Ctx‐PGPe and ‐AGPe connections is available. Regarding Str‐GPe connections, no significant changes in iSPN‐PGPe connections after dopamine depletion have been reported (Cui, Du, et al., 2021; Miguelez et al., 2012). In contrast, the dSPN‐AGPe connection strength has recently been reported to be at least three times as large following dopamine depletion (Cui, Du, et al., 2021). Therefore, the dSPN‐AGPe connections were increased by 200% in this study.

###### Changes in afferent inputs

The firing rate of cortical neurons projecting to striatopallidal neurons for the lesioned condition (6‐hydroxydopamine, or 6‐OHDA) during SWA has been reported to be 1.2 Hz (Mallet et al., 2006). In lesioned (6‐OHDA) rats, iSPNs and dSPNs exhibited higher firing rates of 1.20 and 0.84 Hz, respectively.

During cortical activation, both cortical and striatal activities in the Parkinsonian state show distinct beta‐band oscillation in rats, and the phase of the striatal beta oscillation is the antiphase of the cortical beta oscillation (Mallet et al., 2006; Sharott et al., 2017). Thus, ϕ_Ctx_ was set to zero, and ϕ_Str_ was set to *π*. The amplitudes, *A*_Ctx_ and *A*_Str_, were set to 0.6. The firing rate of iSPN in lesioned rats increased to 2.80 spikes/s, whereas that of dSPN did not change from 1.15 spikes/s (Sharott et al., 2017).

###### Changes in cellular properties

In the Parkinsonian state, the membrane excitability of GPe neurons was enhanced (Kovaleski et al., 2020); however, the detailed mechanism is not yet known. Here, we hypothesized that persistent sodium and potassium KCNQ currents are influenced by dopamine concentration (Fujita et al., 2012). Therefore, for the Parkinsonian condition, g_NaP_ and g_KCNQ_ were increased and decreased by 30%, respectively. This change resulted in a 50% increase in the autonomous firing rate of GPe neurons. In slices, the firing rate changes presumably reflect cellular adaptations rather than a loss in acute dopamine modulation, which is likely to be low in brain slices. These changes are summarized in Table 4.

**TABLE 4:** Effect of dopamine.

Dopamine modulation	
*U*_STN‐GPe_, *U*_GPe‐STN_, *U*_Ctx‐*_, *U*_iSPN‐PGPe_	×0.5
*U*_dSPN‐AGPe_	×1.5
Dopamine depletion	
*g*_GABA_^GPe‐STN^	×1.5
*g*_GABA_^GPe‐GPe^	×1.4
*g*_AMPA_^STN‐PGPe^	×0.7
*g*_AMPA_^Ctx‐STN^	×0.5
*g*_GABA_^dSPN‐AGPe^	×3.0
*R*_base,iStr_	SWA: 0.35 → 1.2 *s*_1_ ACT: 0.5 *s*_i_ → 2.8*s*_i_
*R*_base,dStr_	SWA: 0.3 → 0.8 *s*_d_ ACT: 1.0 *s*_d_ → 1.0 *s*_d_
*A*_Ctx_, *A*_Str_	SWA: *A*_Ctx_ 1.0 → 0.9, *A*_Str_ 0.8 → 1.0 ACT: *A*_Ctx_ 0.0 → 0.6, *A*_Str_ 0.0 → 1.0
*g*_NaP_	×1.3
*g*_KCNQ_	×0.7

#### 2.1.6 Numerical simulations

We conducted numerical simulations of our STN‐GPe network model to reproduce SWA in rats under normal conditions. The parameters to be explored are summarized in Table 5. The network activity satisfied under these conditions consisted of STN, PGPe, and AGPe neuron firing rates of approximately 15, 20, and <5 spikes/s, respectively (Abdi et al., 2015; Mallet et al., 2008). In addition, the phase relations to cortical oscillations were in‐phase for all neuron types.

**TABLE 5:** Parameters to be explored.

Connectivity bias (*k*_1_, *k*_2_, *k*_3_)	0.5, 1, 2 for all; 0.1–0.5 for *k*_2_ and *k*_3_
The number of cortical afferent inputs (*N*_Ctx‐STN_)	0–100 (in 10 increments)
The number of striatal afferent inputs (*N*_Str‐GPe_)	0–1000 (in 100 increments)
Relative amplitudes (*A*_Ctx_, *A*_Str_)	0.2–1.0 (in 0.2 increments)
Topographic connectivity	Topographic or random
Scaling parameters for background inputs (*s*_i_ and *s*_d_)	0–1.0 (in 0.1 increments)
*g*_Ctx‐PGPe_, *g*_Ctx‐AGPe_	0.5–1.5 (in 0.25 increments)

In the first phase of parameter exploration, we evaluated the connectivity biases (*k*_1_, *k*_2_, and *k*_3_) and the number of afferent connections (*N*_Ctx‐STN_ and *N*_Str‐GPe_) simultaneously in a brute‐force manner, setting the amplitudes to a fixed value (0.5). In the second phase, only the amplitudes (*A*_Ctx_ and *A*_Str_) were varied, while the parameters were fixed to the values obtained in the previous phase. Then, the first phase was repeated. If the parameter values converged, they were regarded as the optimal values for this model.

Using the obtained model, we investigated network activities under normal and Parkinsonian conditions, under which, the states of SWA and ACT were examined. These different conditions were implemented by changing the parameter values based on experimental observations (Table 4). Finally, the scaling parameters for striatal firing rates *s*_1_ and *s*_2_ were varied for the Parkinsonian SWA, whereas the cortical firing rates for the normal and Parkinsonian ACT condition. The procedure is summarized as a scheme in Figure 1C.

## 3 RESULTS

We constructed a computational model of the STN‐GPe network to investigate the influence of network properties on the activity (Figure 1A). While many parameters, such as synaptic conductances, were experimentally measured, some parameters were still unknown. Therefore, these parameters and the connectivity between nuclei (Figure 1B) were explored so that the model could reproduce the firing characteristics. Firstly, this parameter exploration was conducted for the normal SWA condition. Then, the attempt to reproduce the Parkinsonian SWA was made by adjusting the experimentally obtained parameter values as necessary. For the cortical ACT conditions, similar attempts were made (Figure 1C). Parameter exploration for those conditions was conducted based on the experimental results on firing characteristics such as firing rates under those conditions.

### Behavior of model neurons and synapses

**FIGURE 2:**
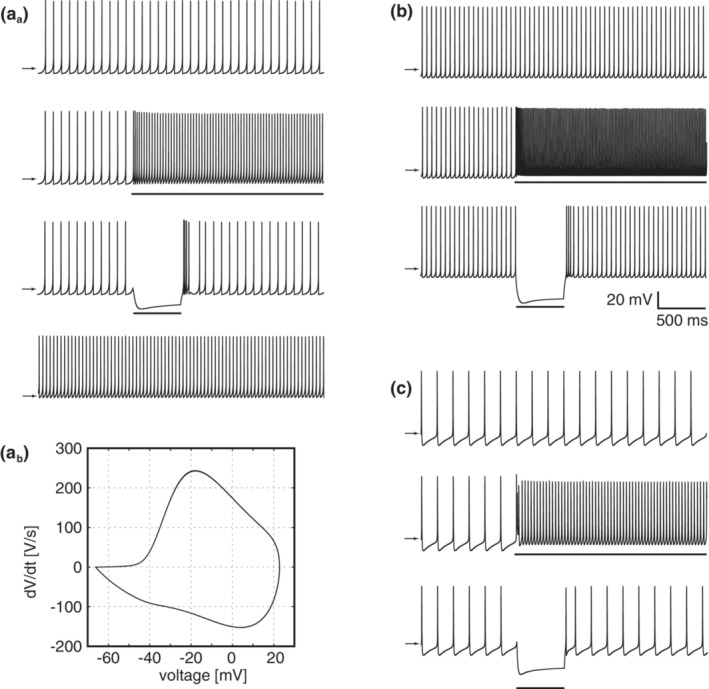
Behavior of model neurons. (A_a_) Spiking activity of a model STN neuron. From the top, autonomous spiking, response to current injection with 40 pA and −50 pA, and autonomous spiking when high‐voltage activated calcium channels were less activated. (A_b_) Phase plot of autonomous spiking of a model STN neuron. (B) Spiking activity of a model prototypic pallidal neuron. From the top, autonomous spiking, response to current injections of 100 pA, and −100 pA. (C) Spiking activity of a model arkypallidal neuron. Similar to (b), but the bottom displays the response to current injection at −80 pA. STN, subthalamic nucleus.

Before constructing the network model, we first constructed single neuron models of the STN, PGPe, and AGPe neurons and synapse models (see Section 2). Using a differential evolution algorithm‐based parameter optimization technique (Storn & Price, 1997), we explored parameter values that reproduced experimentally measured electrophysiological properties (Abdi et al., 2015; Ramanathan et al., 2008). The obtained properties and parameters are listed in Table 1. Moreover, the autonomous activities and responses to current injection are shown in Figure 2A–C. According to the obtained score in Table 1, the AP threshold score for the STN neuron model was larger than the others (a larger score representing a worse score). Reproducing instantaneous generation of action potentials requires an additional compartment of the axon initial segment to the soma. This discrepancy is presumably because the model is the single compartment model. The STN neuron model exhibited a characteristic rebound burst after release from prolonged hyperpolarization (Figure 2A_a_). The PGPe neuron model exhibited high‐frequency autonomous firing, whereas the AGPe neuron model exhibited low‐frequency firing (Figure 2B,C). Furthermore, both models reproduced characteristics of those neuron types, such as the sag potential due to the HCN channels. Thus, although within the range of single‐compartment models, all models represented the characteristics of the corresponding neuron types well.

**FIGURE 3:**
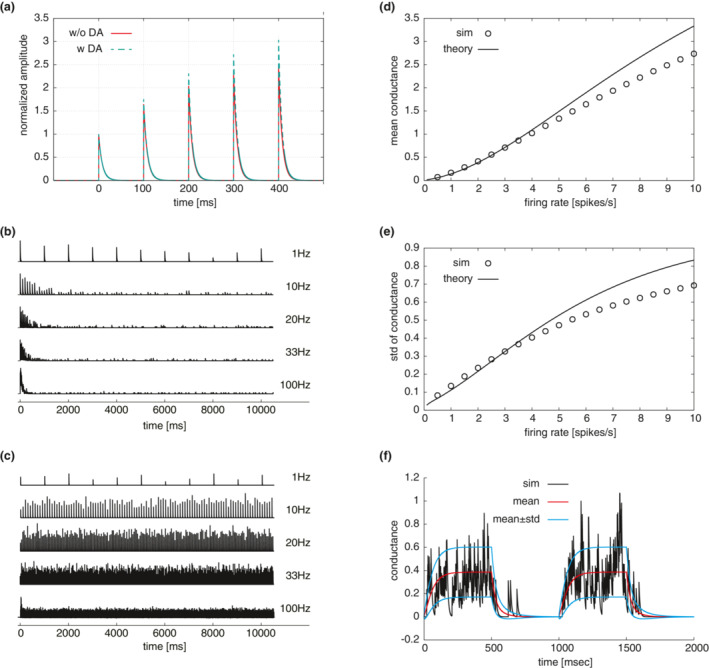
Behavior of model synapses. (A) Time courses of an Str‐GPe synapse responding to repetitive stimulation with 100‐ms intervals. Amplitudes were normalized by the first pulse. The red indicates the response for the control condition, whereas the green mimics the dopamine‐application condition by setting the release probability to the half‐maximal value (*U* = 0.07). (B, C) Effective state ratios (relative conductances to the maximal conductances) of GPe‐STN (B) and STN‐GPe (C) conductances associated with presynaptic firing of 1–100 Hz. (D, E) Comparison of the point conductance approximation with numerical simulation. Simulation data (circles, ‘sim’) were obtained by summation of conductances of *N* synapses receiving independent Poisson spike trains (*N* = 100) while theoretical approximation (solid line, ‘theory’) was calculated with the approximation (see Section 2). The parameters of a synapse model were set to the values for the Str‐GPe synapse in Table 1. Dependence of the mean, *G*_X_^0^ (D) and the standard deviation, *σ* = (*D*_X_ *τ*_*e*_/2)^1/2^ (E) on the presynaptic firing rate, *R*_X_. (F) Comparison of the point conductance approximation with numerical simulation in the case of SWA. The firing rate, *R*_X_(*t*) changed based on the SWA condition. The red and blue lines were obtained by theoretical approximation.

It has been reported that synapses in the STN‐GPe circuit show specific dynamics depending on the type of connection (Atherton et al., 2013; Hanson & Jaeger, 2002). Each type of synapse was modeled based on the depressing/facilitating synapse model (Tsodyks & Markram, 1997). Figure 3A shows the time course of the model responses obtained by adjusting the parameters (*τ*_*r*_, *τ*_u_, and *U*) to reproduce the profiles of the amplitudes in the case of repetitive activation for the Str‐GPe synapse (Miguelez et al., 2012). The synapses in the STN‐GPe circuit, especially the GPe‐STN synapses, exhibit highly stochastic behavior (Atherton et al., 2013). Therefore, for the models of AMPA (α‐amino‐3‐hydroxy‐5‐methyl‐4‐isoxazole propionic acid)‐ and GABA_A_‐mediated synapses, we modified a deterministic depressing synapse model (Tsodyks & Markram, 1997) to implement stochastic neurotransmitter release (Loebel et al., 2009). The time courses of the relative conductance (open probability) of GABAergic GPe‐STN and glutamatergic STN‐GPe synapses for various input frequencies are illustrated in Figure 3B,C, respectively. Due to the large recovery time constant of the GPe‐STN synapses, presynaptic resources could not be sufficiently recovered. Consequently, the relative conductance was significantly reduced. The reliability and normalized conductance of the synapse model are summarized in Table 2. For higher frequency inputs, the reliability and normalized conductance dropped dramatically. The afferent inputs from the cortex and striatum to the STN‐GPe were modeled as the point conductance (Destexhe et al., 2001) to reduce computational costs (see Section 2). The average conductance, *G*_X_^0^, and diffusion coefficient, *D*_X_, could be approximately derived from the synapse model under the assumption that presynaptic spiking activity obeys the Poisson process at a low rate. In Figure 3D,E, the comparison between the simulation results of multiple synapses activated by independent Poisson spike trains and theoretical approximation (the point‐conductance approximation) for facilitating Str‐GPe synapses is shown to confirm whether the approximation holds. As shown, the approximation is in good agreement with the simulation results of the summed conductance of *N*(=100) synapses for a low firing rate (<4 spikes/s). Furthermore, the point conductance approximation held not only for a fixed firing rate, but also for a slowly shifting firing rate. Figure 3F illustrates that the mean and standard deviation of the point conductance approximation characterize the behavior of synaptic dynamics during SWA. Therefore, we used the point conductance model for the afferent inputs from the cortex and striatum.

### 3.2 Impact of connectivity patterns on neural activity

**FIGURE 4:**
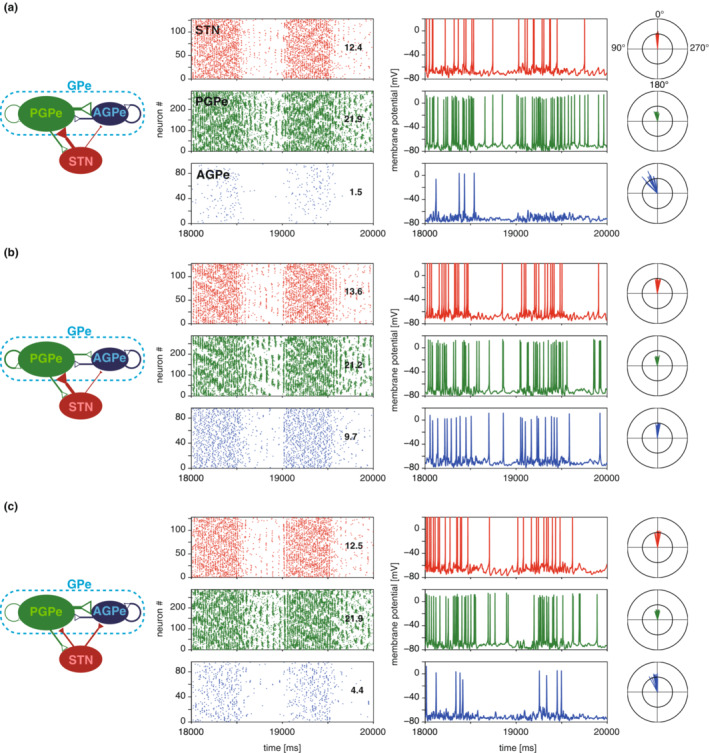
Impact of connection biases on neural activity in the normal slow wave activity condition. Connection structure, network activity (raster displays), membrane potential time courses, and phase of spikes in cortical SWA. Colors correspond to the neuron types in the network structure (left). Figures in the raster displays show average firing rates. The 0° spike phase represents the peak of the fundamental harmonic of SWA. (A) The best network model such that the model satisfies the firing rates and the phase relationship described at “Numerical simulations” in Section 2. The connectivity biases, *k*_1_, *k*_2_, and *k*_3_ = 2, 0.1, and 0.1, respectively. (B) The activity in the network with no bias between the PGPe–PGPe and PGPe–AGPe connections (*k*_1_, *k*_2_, *k*_3_ = 2, 1, 0.1). Only the bias between the PGPe–PGPe and PGPe–AGPe connections was varied from the best configuration shown in (A) (from *k*_2_ = 0.1–1). (c) The activity in the network with no bias between STN‐PGPe and STN‐AGPe connections was varied (from *k*_1_ = 1, 0.1, 0.1). Only the bias between the STN‐PGPe and STN‐AGPe connections was varied from the best configuration shown in (A) (from *k*_1_ = 2 to 1).

Next, we constructed an STN‐GPe network model using the above neuron models. In general, network activity is greatly influenced by the connectivity within the network, the strengths of those connections, and the characteristics of afferent inputs to the network. For the STN‐GPe, since connection strengths have been investigated by several electrophysiological and optogenetic studies (Chu et al., 2017; Cui, Du, et al., 2021; Fan et al., 2012; Miguelez et al., 2012; Pamukcu et al., 2020), those results were used in the model. Therefore, to obtain the condition that aims to reproduce the activity under the normal SWA state, we explored appropriate connectivity between the network (e.g., connectivity between the three cell populations) and characteristics of afferent inputs (e.g., inputs from the cortex and the striatum). In the process of searching for appropriate parameters, we searched by combining connectivity biases (*k*1, *k*_2_, and *k*_3_, in Figure 1B), the number of afferent synapses (*N*_Ctx‐STN_ and *N*_Str‐GPe_), and the amplitudes of SWA inputs (*A*_Ctx_ and *A*_Str_). Figure 4 illustrates how network structure influences network activity, firing rates, and patterns during the SWA state in the dopamine‐intact condition. In Figure 4, the impact of connection biases from STN to PGPe or AGPe (red) and from PGPe to PGPe or AGPe (green) are compared. The network configuration shown in Figure 4A was optimal for satisfying the firing rate criteria of rats (Abdi et al., 2015; Mallet et al., 2008) as a result of the parameter exploration. The parameters for this network were as follows: connection bias parameters, *k*_1_, *k*_2_, and *k*_3_ were 2, 0.1, and 0.1, respectively; the number of cortical and striatal connections, *N*_Ctx‐STN_ and *N*_Str‐GPe_ were 60 and 600, respectively; and amplitudes of SWA, *A*_Ctx_ and *A*_Str_ were 1.0 and 0.8, respectively. In this network, the firing phases of each neuron class in cortical SWA were reproduced. The PGPe firings were weakly phase‐locked to the peak of SWA, while the AGPe firings were tightly phase‐locked. To confirm how the network activity is influenced when the bias is varied from the configuration of Figure 4A,B demonstrates that the connections from PGPe to AGPe or PGPe had no connection selectivity (*k*_2_ = 1) instead of biased connectivity (*k*_2_ = 0.1) as is in Figure 4A. This network structure resulted in an elevation in AGPe firing rates, indicating that intensive inhibitory connections from PGPe were important for controlling the AGPe firing rate. Similarly, in the network shown in Figure 4C, the connections from the STN to GPe had no connection selectivity (*k*_1_ = 1) instead of biased connectivity (*k*_1_ = 2). The AGPe activity was elevated here, as well. However, in both cases, the firing rates of the STN and PGPe were not significantly affected. These results suggest that the neural activity of AGPe neurons is susceptible to changes in the network structure, whereas STN and PGPe activity remained the same. It should be noted that a change in the connection bias from AGPe to GPe (*k*_3_) had no influence on STN‐GPe activity (data not shown).

### 3.3 Impact of afferent input properties

**FIGURE 5:**
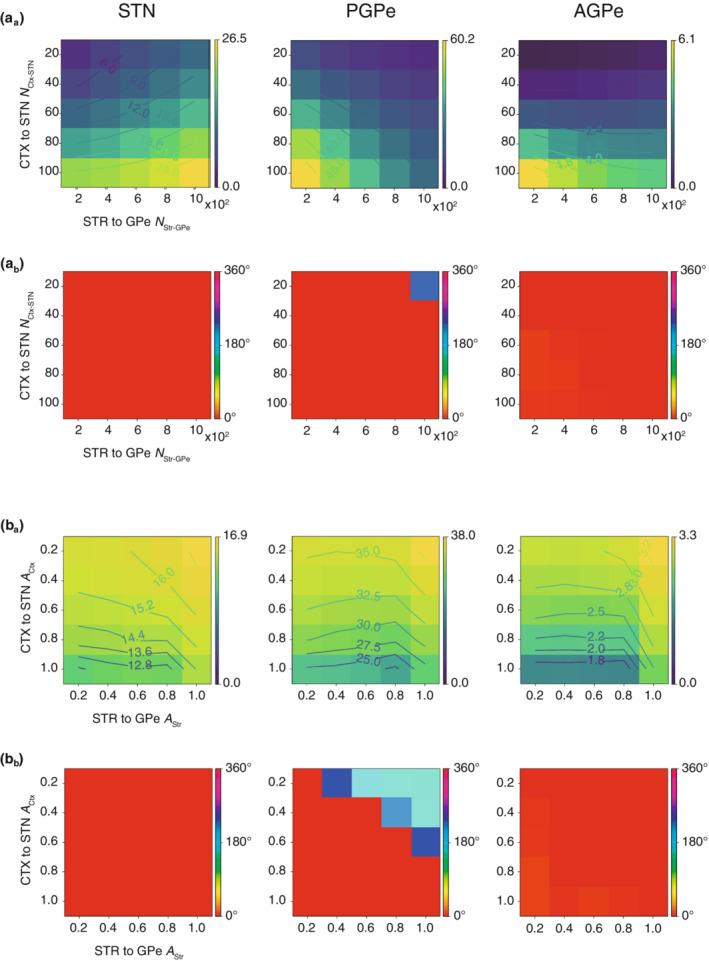
Dependence of neural activity on firing rates and amplitudes of the afferent inputs. (A) Dependence on the connection numbers of the cortical and striatal afferent inputs, *N*_Ctx‐STN_ and *N*_Str‐GPe_. The amplitudes, *A*_Ctx_ and *A*_Str_, were set to the optimal values (1.0 and 0.8, respectively). (A_a_) Colormaps of firing rates of each neuron type. The color scale represents the range from 0 to the maximal values. (A_b_) Colormaps of firing phases of each neuron type. The color scale represents the phase relative to the peak of SWA. (B) Dependence on amplitudes of the cortical and striatal afferent inputs, *A*_Ctx_ and *A*_Str_. The base firing rates, *N*_Ctx‐STN_ and *N*_Str‐GPe_, were set to the optimal values (60 and 600, respectively). (B_a_) Colormaps of firing rates of each neuron type, similar to (A_a_). (B_b_) Colormaps of firing phases of each neuron type, similar to (A_b_).

To reproduce the STN‐GPe activity during SWA, the characteristics of afferent inputs must be determined. Since the characteristics are described by the number of connections and amplitudes of cortical and striatal inputs, the effects of those properties on neural activity are examined. Figure 5A_a_,A_b_ show the dependence of firing rates and phases of those neuron types on connection numbers (*N*_Ctx‐STN_, *N*_Str‐GPe_) under the condition that the other parameters were set to the best values found in Figure 4A. As shown in Figure 5A_a_, STN activity increased with cortical and striatal inputs because cortical inputs directly excite the STN, whereas striatal inputs cause disinhibition of PGPe neurons. Although PGPe and AGPe activity were influenced by cortical and striatal inputs, the effect of the striatal inputs was stronger for PGPe than AGPe. Regarding phase relations to SWA, the only neurons affected were PGPe neurons under the conditions of weak cortical and strong striatal inputs (Figure 5A_b_). The amplitudes of cortical and striatal rhythmicity (*A*_Ctx_ and *A*_Str_) also had a considerable impact. Figure 5B demonstrates how the amplitudes influence STN‐GPe activity when the other parameters are set to optimal values. Regarding the dependence of the STN‐GPe firing rates on SWA amplitudes, the amplitude of the cortical SWA had the same effect on all cell types; as the amplitude becomes greater, the firing rates of all cell types become smaller. On the contrary, the amplitude of the striatal input had less effect on PGPe and AGPe (Figure 5B_a_). With respect to the phase relationships to SWA (Figure 4B_b_), the phase of PGPe activity was inverted in the region satisfying *A*_Ctx_ < *A*_Str_ (blue) (Figure 5B_b_). Thus, because afferent inputs had a strong effect on STN‐GPe firing rates and phase relations, we could determine the necessary characteristics of the afferent inputs.

### 3.4 SWA under Parkinsonian conditions

As a result of parameter exploration, we obtained an STN‐GPe network model showing the experimentally observed network activity for the normal SWA (Figure 4A). The policy for reproducing pathological activity (firing rates and phases to SWA) was as follows. First, the experimentally obtained parameter values, such as synaptic conductances in the pathological state (“Dopamine depletion” in Table 4), were applied to the model obtained in the normal state described above, and attempts were made to adjust the other parameters as necessary.

**FIGURE 6:**
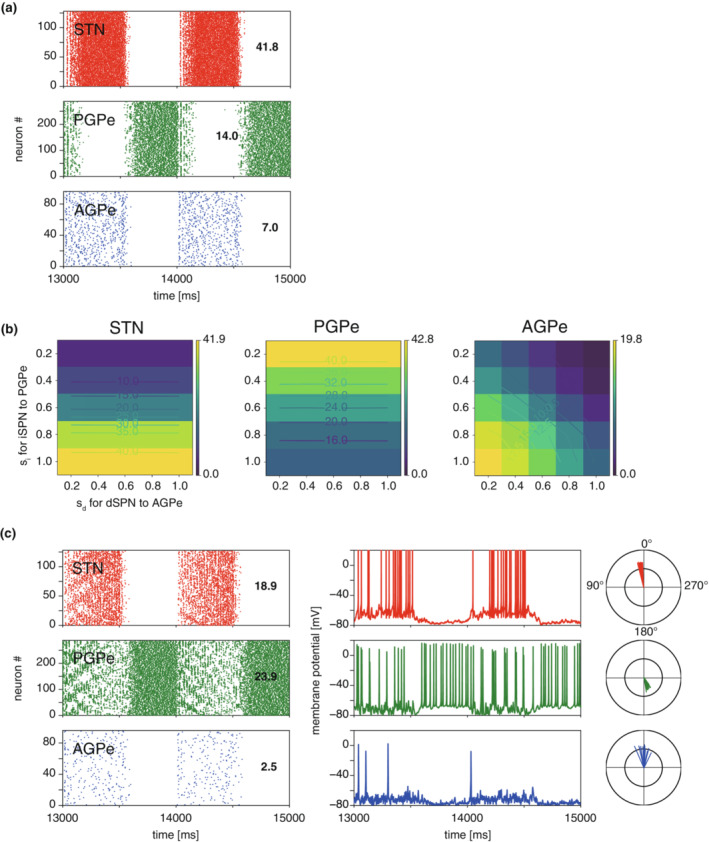
Slow wave activity in the Parkinsonian condition. (A) Network activity when known parameter changes following dopamine depletion (Table 3) were applied to the parameter setting reproducing the normal SWA shown in Figure 4A. The adjustment of striatal firing rates was not applied yet, (i.e., *s*_i_, *s*_d_ = 1.0, 1.0). (B) Dependence on scaling parameters, *s*_i_ and *s*_d_, of STN‐GPe firing rates. Based on the experimentally observed firing rates of STN and GPe neurons, effective firing rates of iSPN and dSPN for striato‐pallidal inputs were explored by introducing the scaling parameters. (C) Resultant network activity during SWA for the Parkinsonian condition after adjustment of model parameters. Spike raster displays, membrane potential time courses, and phases of spikes to SWA, similar to Figure 4. In addition to the parameter changes summarized in Table 3, the scaling parameters of striatal firing rates, *s*_i_ and *s*_d_, were set to 0.6 and 1.0.

The known alterations caused by chronic dopamine depletion consist of modifications of synaptic strength, afferent input firing rates, and cellular properties (“Dopamine depletion” in Table 4). The resulting network activity obtained by applying such alterations to the model in Figure 4A is shown in Figure 6A. PGPe activity was strongly inhibited by elevated striatal activity. Because the in vivo PGPe firing rate during Parkinsonian SWA is reported to be 18–25 spikes/s (Abdi et al., 2015; Mallet et al., 2008), the synaptic inputs from the striatum in Figure 6A should be too strong. The decrease in synaptic conductance of the intra‐GPe connections and the increase in synaptic conductance of the STN‐GPe connections are possible alternative changes; however, we followed the experimentally observed values for these conductances and fixed those values. Thus, it is necessary to reduce the conductance in order to represent the actual level of synaptic currents by the point conductance approximation. To estimate the actual strengths of the striatal inputs based on GPe activity, scaling parameters for iSPN and dSPN firing rates were introduced to explore the “effective” firing rate for the afferent input models. Figure 6B displays the dependence of neural activity in the STN‐GPe network on the scaling parameters for the iSPN and dSPN firing rates (*s*_*i*_ and *s*_*d*_, respectively). A smaller *s*_i_ (lower “effective” iSPN firing rate) resulted in a higher PGPe firing rate, which caused lower STN activity due to strong inhibition. Furthermore, AGPe activity was influenced by both iSPN and dSPN firing rates. As previously reported (Mallet et al., 2008), the firing rate of STN neurons was approximately 20 spikes/s, and *s*_i_ was set to 0.6. Because the increase in the dSPN firing rate due to dopamine depletion was moderate compared to that of iSPN (Sharott et al., 2017), the dSPN firing rate requires less adjustment than the iSPN firing rate. Therefore, *s*_d_ was set to 1.0. After applying the effective firing rates, the network activity changed as shown in Figure 6C, which is the resulting network activity generated by the “Parkinsonian” conditions of our model. In the Parkinsonian condition, the PGPe neuron firings were antiphase to the SWA of the normal model.

The requirement to adjust striatal firing rates can be attributed to the following two points: the point‐conductance approximation of afferent inputs does not hold for a high firing rate, and the striatal firing rate for the Parkinsonian SWA condition is considerably high. The former is shown in Figure 3D,E. The point conductance approximation tended to overestimate the mean and standard deviation of the summed conductance for the higher mean firing rate (>5 spikes/s). The latter would be evidenced by the following points. Striatal firing rates exhibit a log‐normal‐like broad asymmetric distribution, in which the firing rates of most striatal neurons are very low (**≪**1 spike/s) whereas the remaining neurons discharge at a much higher rate than average (Sharott et al., 2017). In addition, the striatal firing rate increased two‐ to threefold in the Parkinsonian condition (Sharott et al., 2017). In SWA, the firing rate at oscillation peaks increased to almost double the mean firing rate, while it almost vanished at the troughs. Thus, the peak firing rate of “active” striatal neurons during Parkinsonian SWA is considerably high. Taken together, the conductance approximated by the point conductance model should extremely overestimate actual conductance under Parkinsonian SWA conditions.

### 3.5 ACT versus SWA in the normal and Parkinsonian conditions

**FIGURE 7:**
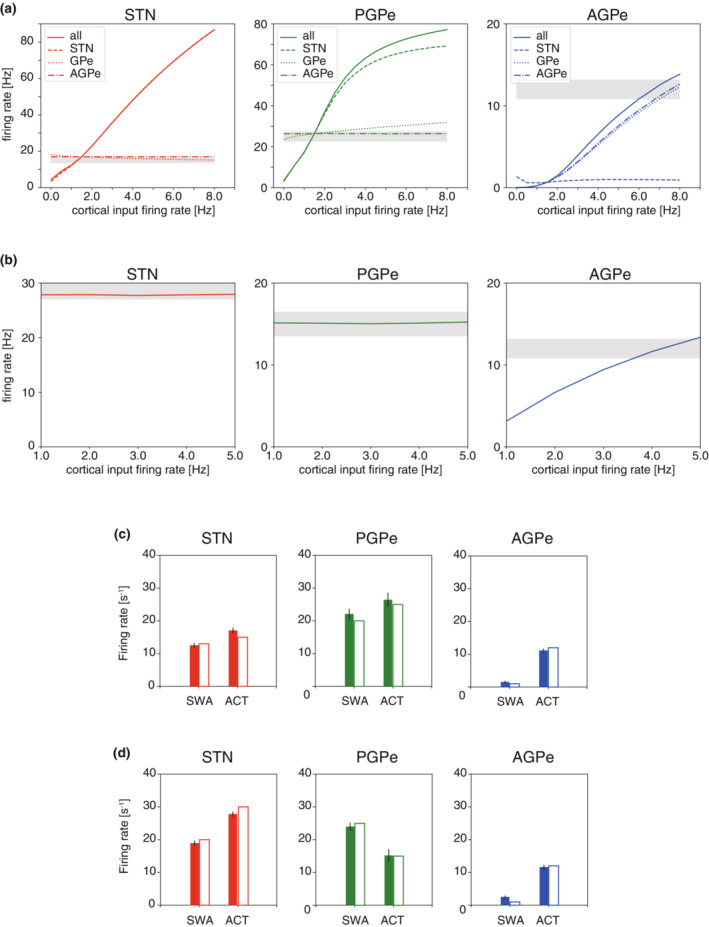
SWA and ACT in the normal and Parkinsonian conditions. (A) Dependence of the firing rates of STN, PGPe, and AGPe in the normal SWA condition on the cortical firing rates. “all,” “STN,” “GPe,” and “AGPe” in legends indicate the cases where the cortical firing rate corresponding to the afferent to all neurons (STN, PGPe, AGPe), STN, PGPe, and AGPe was varied, respectively. The cortical firing rate for the rest of the afferents was kept at 1.5 spikes/s. The gray zones represent experimentally measured rates (mean ± 10%; STN: 13.5–16.5, PGP: 22.5–27.5, AGPe: 10.8–13.2). (B) Similar to (A), but in the Parkinsonian ACT condition. The cortical firing rate for the afferent to AGPe was varied, whereas those for the afferents to STN and PGPe were kept to 2.5 spikes/s. The gray zones are as follows; STN: 37–33, PGPe: 13.5–16.5, AGPe: 10.8–13.2. (C) Comparison of firing rates between SWA and ACT under normal conditions. The filled bars and open bars represent the simulation and experimental results, respectively. The experimental data were taken from Mallet et al. (2012) for the STN and PGPe and Abdi et al. (2015) for the AGPe. The vertical line on the filled bar indicates the standard deviation. (D) Comparison of firing rates between the SWA and ACT for the Parkinsonian condition, similar to (B).

STN‐GPe activity in the ACT state was demonstrated by adjusting cortical and striatal afferent inputs. In simulations of ACT conditions, the cortical and striatal input firing rates and amplitudes were changed, while the other parameters remained the same, as shown in Table 4. Under normal conditions, the inputs displayed no rhythmicity when the amplitude parameters were set to 0 and the intensity of the inputs was adjusted by the base firing rates (see Section 2). Firstly, in the case where the cortical firing rate was set to 1.5 spikes/s, which has been reported for the normal SWA condition (Mallet et al., 2012), the firing characteristics of the STN‐GPe were not satisfied (data not shown). Therefore, I explored the appropriate firing rate of cortical afferents for the normal ACT condition. Figure 7A displays the firing rates of the three nuclei when the cortical firing rate from the cortex to all, only STN, GPe (PGPe and AGPe), and AGPe were varied. When the firing rate of the afferent to STN was increased (all and STN), the activities of STN and PGPe were excessively elevated. To satisfy the firing characteristics of all nuclei, it was necessary to increase the firing rate of only the afferent to AGPe (the rate of the afferents to STN and PGPe was set to 1.5, whereas the rate of the afferent to AGPe was 6.0 spikes/s). Similarly, the firing rates of the cortical afferents were adjusted for the Parkinsonian ACT condition after applying the parameter changes caused by chronic dopamine depletion. Figure 7B illustrates the dependence of STN‐GPe firing rates on the cortical firing rate of the afferent to AGPe. In this case, as well, the firing rate of the other cortical afferent was kept constant (2.5 spikes/s). These results indicate that the cortical activity of AGPe is different from that of the other nuclei (STN and PGPe) under ACT conditions. The summary of the simulated activity of the STN‐GPe network for the normal and Parkinsonian conditions is in Figure 7C,D, respectively. The firing rates of all neuron types under normal conditions were elevated for ACT compared to those of SWA, whereas PGPe neurons showed the opposite change for the Parkinsonian condition. Thus, the proposed STN‐GPe network model could generally reproduce experimentally observed firing characteristics of all nuclei by adjusting afferent input properties. Furthermore, the obtained results were not sensitive to the parameter changes because the firing rates (when functioning as the parameters) changed gradually and not abruptly, as shown in Figure 5A,B. Only the phase relationship with the SWA was exceptional; a slight change in amplitude *A*_Str_ or *A*_Ctx_ caused the phase to shift from in‐phase to anti‐phase (Figure 5B_b_). This low sensitivity of firing activity to the parameters was present for the connectivity biases (*k*_1_, *k*_2_, and *k*_3_), numbers of inputs (*N*_Ctx‐STN_ and *N*_Str‐GPe_), and amplitudes of those inputs (*A*_Ctx_ and *A*_Str_).

### 3.6 Factors of transition from normal to pathological activity

**FIGURE 8:**
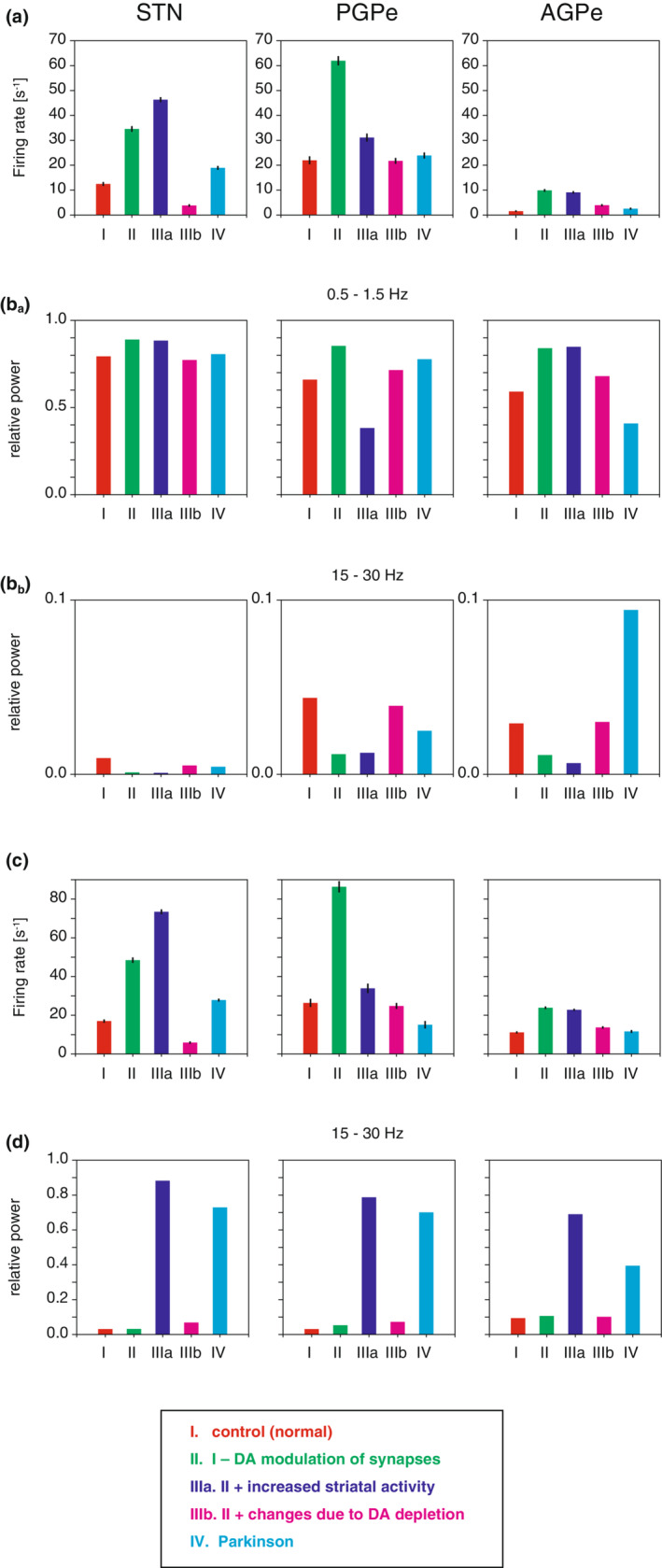
The factors that induce pathological neural activity. (A) Firing rates for various conditions during SWA. Colors correspond to the condition from I to IV in the legend. (B) Relative power of spiking activity over a neural population in the 0.5–1.5 Hz frequency band during SWA. The relative power was obtained by normalizing the power within the range by the total power from 0–100 Hz. (B_a_) Relative power of spiking activity over a neural population in the 0.5–1.5 Hz frequency band. (B_b_) Relative power of spiking activity over a neural population in the 15–30 Hz. (C) Firing rates for various conditions during ACT. Only the conditions where the afferent inputs were kept in the normal condition were investigated. (D) Similar to B_b_, but during ACT.

Many changes triggered by dopamine depletion are involved in the transition from the normal to pathological firing mode. We examined these changes more closely to identify the most significant factor of transition. The factors included (i) loss of dopaminergic modulation of synaptic transmission, (ii) markedly elevated iSPN input to the GPe, and (iii) long‐term potentiation/depression (LTP/LTD) of synaptic connections and changes in cellular properties caused by chronic dopamine depletion. The firing rates of the neuron types under various conditions during SWA are shown in Figure 8A. Loss of dopaminergic modulation of synaptic transmission (condition II, Figure 8) demonstrated a significant impact on all neuron types, leading to large increases in the firing rates in comparison with the control condition (condition I). In addition, the elevated striatal firing rate (condition IIIa) strongly inhibited PGPe activity, while it enhanced STN and AGPe activity due to the disinhibition of inputs from the PGPe. If LTP/LTD, cellular property changes, and as the dopaminergic modulation of synaptic transmission occurred (condition IIIb), the activity of all nuclei, especially of the STN, dramatically decreased. With regard to the STN firing rate, increased striatal activity and LTP/LTD had contrasting effects. Thus, these changes could cancel out the effects, resulting in no overall change.

No significant changes were observed in the power of the frequency bands during SWA (Figure 8B). Because the network received SWA inputs from the cortex and striatum, the corresponding SWA frequency band (0.5–1.5 Hz) comprised the majority of the total power (0–100 Hz) (Figure 8B_a_). Notably, LTP/LTD amplified the beta‐band activity for all neuron types (Figure 8B_b_).

To observe how these changes brought on by dopamine depletion affect the network activity during ACT, we simulated the conditions where the afferent inputs were maintained under normal conditions (conditions I, II, and IIIb, Figure 8). As with SWA, the loss of dopaminergic modulation on synapses caused notable increases in firing rates, while LTP/LTD of synaptic connections canceled these increases (Figure 8C). At the same time, the relative power of the beta‐band component increased, even though the inputs contained no beta‐band component (condition IIIb), suggesting that the network developed intrinsic properties to enhance beta‐band activity (Figure 8D).

Thus, changes caused by chronic dopamine depletion, especially LTP/LTD in connections between nuclei (condition IIIb), would compensate for the effect of loss of dopaminergic modulation on firing rates in the STN‐GPe network (condition II). Instead, such a compensatory effect would bring excessive sensitivity to the beta‐band frequency.

## 4 DISCUSSION

Dopamine depletion is responsible for the pathological neural state and symptoms of Parkinson's disease; however, the mechanism underlying this condition is not straightforward. The confusion in understanding this process is likely because the loss of dopaminergic action on neurons and synapses triggers compensatory actions in the basal ganglia at the early stage, and the resultant change could later influence the cortico‐basal gangliothalamic loop. The STN‐GPe network is an original and driving force of this process in the loop. Therefore, the functional organization of the STN‐GPe network, particularly the factors that determine the normal and pathological states, must be understood. Using a computational approach, a model that can imitate both normal and pathological neural states is necessary.

Thus, the present simulation study explored the plausible structure of the STN‐GPe network, utilizing the resulting evidence of the network to explore normal and pathological conditions. The parameters concerning connectivity among PGPe, AGPe, and STN neurons were evaluated and found to have a great impact on STN‐GPe activity. The characteristics of the obtained network were massive innervation from the STN to PGPe and from the PGPe to AGPe. These characteristics were consistent with recent experimental study using whole‐cell recording and optogenetics (Ketzef & Silberberg, 2021). The charcteristics were similar to those of the network in a previous computational study (Nevado‐Holgado et al., 2014), but excitatory connections differed. This could be because the model in the previous study incorporated excitatory thalamic inputs (Bevan et al., 1995; Yasukawa et al., 2004) and was based on firing rate dynamics. Further, the network structure would be consistent with the model for locomotion in which inhibition from PGPe to AGPe and excitation from STN to PGPe would play important roles in GO/No‐GO signaling in locomotion (Aristieta et al., 2021). More detailed connection patterns than that obtained in the present study should be necessary for inter‐ and intraconnections between the cell populations.

The model parameters (such as the connectivity biases and the number of connections) were adjusted based on the firing rates obtained from experiments with rats (Abdi et al., 2015). Therefore, the obtained network structure should represent the STN‐GPe network of rats. The firing rates of these neurons are much higher in monkeys than in rats, especially in the GPe neurons, where this rate is reported to be between 55 (Elias et al., 2007) to 65 spikes/s (Tachibana et al., 2011). In mice, the firing rates of these neuron types are similar to those of rats, but slightly lower; firing rates in the PGPe neurons are reported as 16.7–16.8 spikes/s in mice (Abecassis et al., 2020; Kovaleski et al., 2020). If membrane properties of these neuron types are similar across species, the different rates may be due to the relative numbers of cells because the ratio of STN neurons to GPe neurons is larger in monkeys than in rats and mice; subsequently, more inhibitory activity would be required to balance excitatory STN activity (Hardman et al., 2002). The PGPe neurons in rats show similar firing properties to those in monkeys with high firing rates and pauses (Abdi et al., 2015). However, pauses of GPe neurons have not been reported in rats or mice. This is presumably because for spike trains with low firing rates in these animals, it is not clear whether or not the intervals without spikes are pauses. Similarly, AGPe neurons in rats show firing rates and patterns similar to those in monkeys, which are characterized as “low‐frequency discharge bursting” neurons. However, analysis of burst firing by identified AGPe neurons in mice or rats has not been well established.

We also found that the obtained network model successfully reproduced the changes in firing activity patterns between SWA and ACT under normal and pathological conditions. It is important to understand the disease's progress to elucidate the mechanism of the pathogenesis. However, it might be difficult to experimentally observe the intermediate state between the normal (condition I in Figure 8) and completed pathological states (condition IV in Figure 8). After dopamine depletion, it is unclear which comes first, an increase in striatal activity (condition IIIa in Figure 8) or changes duet DA depletion (condition IIIb in Figure 8). These changes, in either case, were found to compensate firing rate elevation in STN‐GPe network caused by dopamine depletion. Although it is not known if such compensatory actions are an intrinsic nature of neural system, but similar phenomenon was observed in other neural system (Care et al., 2020). Knowing what is happening during the transition from the normal and the pathological states would help develop treatments for the disease.

The striatum is the main target of innervation by midbrain dopaminergic neurons. Therefore, the changes caused by dopamine depletion are prominent in the striatum. Regarding the change in firing rates of the STN‐GPe network, the increased striatal activity is likely to compensate for the elevated PGPe activity caused by the loss of dopaminergic modulation in synapses within the STN‐GPe (Figure 8A). The resultant synaptic modification in the GPe‐STN and Ctx‐STN can be understood as a remedy for the extremely increased STN firing rate caused by the disinhibition of PGPe (Chu et al., 2017). In exchange for the maintenance of activity level, however, the STN‐GPe network became potentially sensitive to the beta‐band component (Figure 8B_b_). Thus, the STN‐GPe network after dopamine depletion appeared to be ready to resonate the cortical and/or striatal inputs with the exaggerated beta component seen in the Parkinsonian condition. The synaptic modification in the GPe‐STN and Ctx‐STN compensated for most of changes in firing activity caused by loss of dopamine, except the increased power of the beta component in AGPe and increased striatal activity. If the compensatory action is triggered by the elevated striatal inputs, then preventing the increased striatal activity may reduce maladaptation of STN‐GPe network and could prevent increased sensitivity to the beta component in cortical and striatal inputs. Thus, preventing the increased striatal activity could be a potential target for the clinical treatment for the Parkinson's disease.

To obtain the firing activity during the cortical ACT condition, the input firing rate of the afferent to AGPe was adjusted separately from that of STN and PGPe. This implies that the projection from the cortex to AGPe may play an independent functional role from those to STN and PGPe. Recently several studies have reported the functional significance of cortico‐pallidal projection (Abecassis et al., 2020; Grewal et al., 2018; Milardi et al., 2015). It has been reported that AGPe neurons form a distinct circuit with striatal projection neurons in the direct pathway and play a distinct role in movement control (Cui, Du, et al., 2021). Taken together, the cortico‐AGPe pathway and the direct pathway with AGPe might serve as novel pathways in addition to the hyper‐direct, direct, and indirect pathways in the cortico‐basal ganglia‐thalamic loop.

## AUTHOR CONTRIBUTIONS

Design of the work, modeling, simulation, analyses, writing were done by K.K.

## FUNDING INFORMATION

This work was supported by MEXT KAKENHI (grant number 15H05877).

## CONFLICT OF INTEREST STATEMENT

The author declares no competing interests.

## ETHICAL STATEMENT

Since this study is a simulation study and does not include any data of human and animal patients, it does not require ethics committee approval.
